# New Contributions to the Diversity of Hypotrichous Ciliates: Description of a New Genus and Two New Species (Protozoa, Ciliophora)

**DOI:** 10.3389/fmicb.2021.712269

**Published:** 2021-08-05

**Authors:** Jiyang Ma, Tengyue Zhang, Weibo Song, Chen Shao

**Affiliations:** ^1^Institute of Evolution and Marine Biodiversity, Ocean University of China, Qingdao, China; ^2^Laboratory of Protozoological Biodiversity and Evolution in Wetland, College of Life Sciences, Shaanxi Normal University, Xi’an, China; ^3^Department of Zoology, Comenius University in Bratislava, Bratislava, Slovakia; ^4^Laboratory for Marine Biology and Biotechnology, Qingdao National Laboratory for Marine Science and Technology, Qingdao, China

**Keywords:** *Bakuella xianensis* n. sp., molecular phylogeny, morphology, *Wilbertophrya sinica* n. g., n. sp.

## Abstract

Ciliated protists (ciliates) are extremely diverse and play important ecological roles in almost all kinds of habitats. In this study, two new hypotrichs, *Wilbertophrya sinica* n. g. and n. sp. and *Bakuella xianensis* n. sp., from China are investigated. *Wilbertophrya* n. g. can be separated from related genera mainly by the combination of lacking a buccal cirrus, pretransverse cirri, and caudal cirri, while possessing frontoterminal cirri. Analyses based on morphological and molecular data confirm the validity of the species, *W. sinica* n. sp., which is characterized as follows: body 50–115 μm × 15–35 μm *in vivo*; midventral complex comprises four or five cirral pairs only and terminates above mid-body; three frontal, two frontoterminal cirri, and two to four transverse cirri; about 15 macronuclear nodules; colorless cortical granules sparsely distributed. Another new species, *B. xianensis* n. sp., was isolated from a freshwater wetland and is defined as follows: body 115–150 μm × 40–65 μm *in vivo*; about 70 macronuclear nodules; dark-brownish cortical granules in groups; midventral complex comprises 8–12 cirral pairs forming a row that terminates posteriorly in mid-body region and two or three short midventral rows that are continuous with the row of midventral pairs; three frontal, four to six frontoterminal, and three to five fine transverse cirri; three bipolar dorsal kineties. Phylogenetic analyses based on small subunit ribosomal DNA (SSU rDNA) sequence data suggest that the new genus *Wilbertophrya* n. g. belongs to an isolated clade, which might represent an undescribed taxon at the family level, whereas *B. xianensis* n. sp. groups with several congeners and members of other related genera are within the core urostylids.

## Introduction

Hypotrich ciliates are a large, ubiquitous group that play key roles in many ecosystem processes and as model organisms in a wide range of biological studies (Berger, 1999, 2011; Song and Shao, 2017; Chen et al., 2019, 2020; Kim and Min, 2019; Kim et al., 2019; Li et al., 2019; Wang et al., 2019; Gao et al., 2020; Sheng et al., 2020). New hypotrich taxa are continuously being discovered, supporting the notion posited by Foissner et al. (2008) that over 80% of ciliate diversity is still undescribed (Bharti et al., 2019; Hu et al., 2019; Kaur et al., 2019; Moon et al., 2019; Dong et al., 2020; Park et al., 2020; Xu et al., 2020).

Urostylids are one of the most speciose and best-known groups within the subclass Hypotrichia Stein, 1859. Nevertheless, their evolutionary relationships and genus- or family-level definitions remain problematic as recognized in several recent reports (Berger, 2006; Lu et al., 2018, 2020; Luo et al., 2018, 2019; Jung and Berger, 2019; Wang et al., 2021a; Zhang et al., 2020).

Here, we document two hypotrichs from inland China: a novel form that represents a new genus and a new species (*Wilbertophrya sinica* n. g., n. sp.) found in a puddle in a forest in Tibet and a freshwater species, *Bakuella xianensis* n. sp., isolated from a wetland in the Qinling Mountains area near Xi’an. Both species were characterized based on morphological observations of specimens *in vivo* and following silver staining. In addition, their molecular phylogeny was analyzed based on small subunit ribosomal DNA (SSU rDNA) sequence data.

## Materials and Methods

### Sampling, Cultivation, and Morphological Studies

**FIGURE 1:**
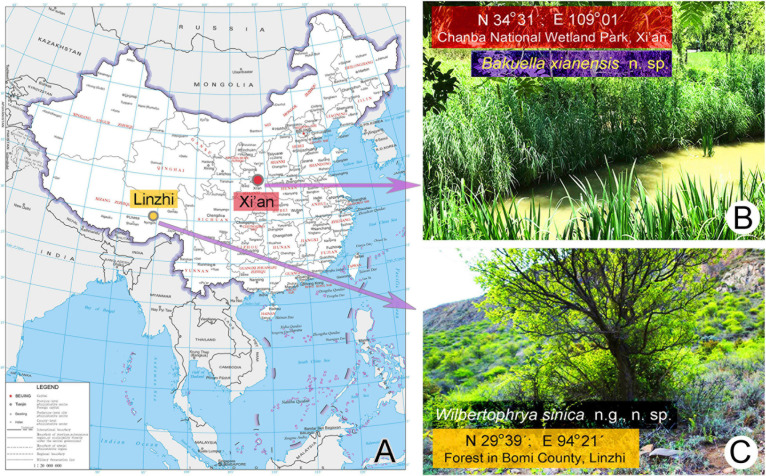
Sample sites. **(A)** The map of China from the MAP WORLD [www.tianditu.gov.cn, drawing review number: GS (2019) 1708] showing the location of the sampling sites. **(B)** Wetland at Chanba National Wetland Park, Xi’an, where *Bakuella xianensis* n. sp. was collected. **(C)** Forest in Bomi County, Linzhi, Tibet, where *Wilbertophrya sinica* n. sp. was collected.

*Wilbertophrya sinica* n. g., n. sp. was collected on April22, 2017, from a small puddle in a forest in Bomi County, Linzhi (29°39′N; 94°21′E), Tibet, where the altitude is about 3,000 m above sea level (Figures 1A,C).

*Bakuella xianensis* n. sp. was collected on May 25, 2019, from a well-vegetated freshwater pond located in Chanba National Wetland Park, Xi’an (34°31′N; 109°01′E), near the junction of the River Ba and River Wei (Figures 1A,B).

Uni-protistan cultures were established in Petri dishes containing rice grains to support the growth of bacteria, which served as a food source for the ciliates. The species was accurately identified based on its morphology *in vivo*. Moreover, no other urostylids morphotypes were present in the protargol preparation. We assure the accuracy of that identification for molecular studies, even though we were unable to establish clonal cultures.

Ciliate cells were observed *in vivo* using bright field and differential interference contrast microscopy. The protargol silver staining method according to Wilbert (1975) was used to reveal the infraciliature. Counts and measurements of stained specimens were performed at a magnification of ×1,000. Drawings of stained specimens were performed at ×1,250 magnification with the aid of a camera lucida. Terminology is mainly according to Berger (2006), and the systematic classification follows Lynn (2008).

### DNA Extraction, PCR Amplification, and Sequencing

Single cells of each species were isolated from cultures, washed several times with distilled water using a micropipette in order to remove potential contamination, and then transferred to 1.5-ml microfuge tubes with a minimum volume of water. Genomic DNA extraction was performed with the DNeasy Blood and Tissue Kit (Qiagen, Hilden, Germany) according to the modified manufacturer’s protocol (1/4 of suggested volume for each solution) (Chen et al., 2019; Lu et al., 2020). PCR amplification of the SSU rDNA was performed using Q5 Hot Start high fidelity DNA polymerase (NEB, Ipswich, MA, United States) to minimize the possibility of amplification errors. One cycle of initial denaturation at 98°C for 30 s, followed by 18 cycles of amplification (98°C, 10 s; 69–51°C touchdown, 30 s; 72°C, 1 min), and another 18 cycles (98°C, 10 s; 51°C, 30 s; 72°C, 1 min), with a final extension of 72°C for 5 min. Sequencing of PCR products was performed bidirectionally on an ABI 3700 sequencer using two PCR primers 18S-F (5′-AAC CTG GTT GAT CCT GCC AGT-3′) and 18S-R (5′-TGA TCC TTC TGC AGG TTC ACC TAC-3′) (Medlin et al., 1988).

### Phylogenetic Analyses and Sequence Comparison With Related Species

**FIGURE 2:**
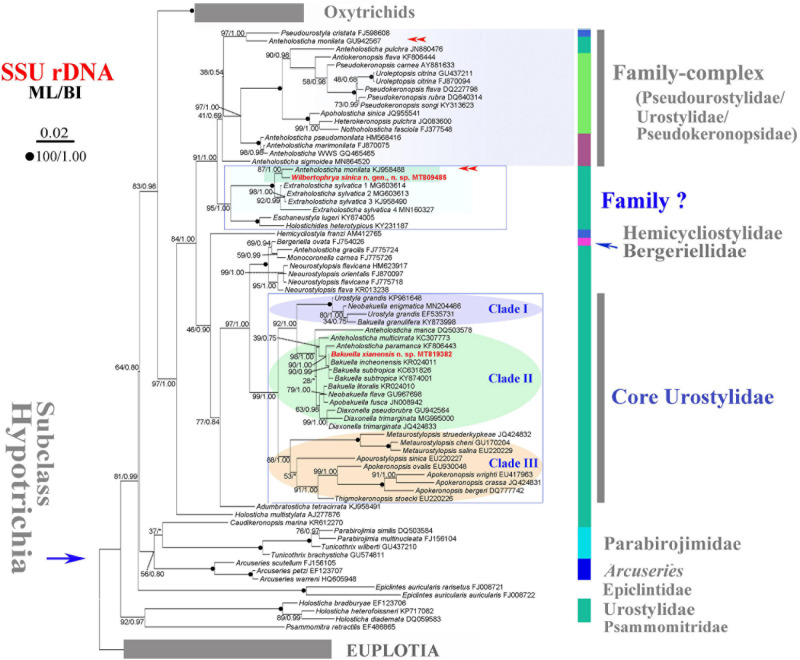
Maximum likelihood (ML) tree inferred from the small subunit ribosomal DNA (SSU rDNA) sequences showing the position of *Wilbertophrya sinica* n. sp. and *Bakuella xianensis* n. sp. (in red) and related species (in rectangular boxes). Numbers near nodes are bootstrap values for maximum likelihood and posterior probability values for Bayesian inference (BI). “*” at nodes indicates disagreement between the two methods. Fully supported (100/1.00) branches are marked with filled circles. Double arrowheads mark the sequences with the same species name, of which one could be misidentified. The scale bar corresponds to 0.02 expected substitutions per site.

In order to perform phylogenetic analyses, the SSU rDNA sequences of *W. sinica* n. g., n. sp. and *B. xianensis* n. sp. were aligned with other related sequences downloaded from the National Center for Biotechnology Information (NCBI) Database (accession numbers shown in Figure 2) on the GUIDANCE2 server^1^ with default parameters (Sela et al., 2015). Representative species of the subclass Euplotia Jankowski, 1979, were selected as the outgroup. The aligned sequences were manually edited using the program BioEdit 7.2.5 (Hall, 1999), resulting in a final alignment with 1,772 sites. Both maximum likelihood (ML) and Bayesian inference (BI) analyses were performed on the final alignment. The ML analysis was performed with 1,000 rapid bootstrap replicates using RAxML-HPC2 on XSEDE v8.2.12 (Stamatakis, 2014) on CIPRES Science Gateway with the GTRGAMMA model (Miller et al., 2010). The BI analysis was computed by MrBayes on XSEDE 3.2.6 (Ronquist et al., 2012) on CIPRES Science Gateway with the GTR + I + G model selected by MrModeltest 2.2 (Nylander, 2004). Markov chain Monte Carlo (MCMC) simulations were run for 10,000,000 generations with sampling every 100 generations. The first 10,000 trees were discarded as burn-in. Seaview v.4.3.3 (Gouy et al., 2010) and MEGA v.6 (Tamura et al., 2011) were used to visualize the tree topologies.

The SSU rDNA sequences were aligned and manually modified (trimming ends and removing identical nucleotides) with BioEdit 7.0.5.2 (Hall, 1999).

## Results

ZooBank registration.

Present work: urn:lsid:zoobank.org:pub:C0BE1314-C4 93-430C-9903-8E1758A1F983

Subclass Hypotrichia Stein, 1859

Order Urostylida Jankowski, 1979

Genus *Wilbertophrya* n. g.

### Description of *Wilbertophrya* n. g.

ZooBank registration.

*Wilbertophrya* n. g.: urn:lsid:zoobank.org:act:61BC94E 2-17BA-48D8-9219-921267C0919B

*Diagnosis:* Urostylid with a continuous adoral zone; three clearly differentiated frontal cirri; midventral complex composed only of cirral pairs arranged in a zigzag pattern; frontoterminal and transverse cirri present; buccal, pretransverse, and caudal cirri absent; one marginal row on each side, the anterior end of the left marginal row not curved rightward; three dorsal kineties.

*Type species: W. sinica* n. sp.

*Dedication:* This new genus is named after the eminent ciliatologist Prof. Norbert Wilbert, University of Bonn, Germany, in recognition of his life-long contributions to the taxonomy of ciliates. Feminine gender.

### Description of *Wilbertophrya sinica* n. sp.

ZooBank registration.

*Wilbertophrya sinica* n. sp.: urn:lsid:zoobank.org:act:F2D0E277-FB7A-493F-90AC-8D9875AC347F

*Diagnosis:* Body 50–115 μm × 15–35 μm *in vivo*, elliptical in outline; 13–19 macronuclear nodules; contractile vacuole located ahead of mid-body; cortical granules colorless, about 1 μm across, irregularly distributed; about 15–18 adoral membranelles; four or five midventral pairs in anterior half of cell; three frontal, two frontoterminal, and two to four transverse cirri; the left marginal row is composed of 13–21 cirri, and right marginal row is composed of 14–25 cirri; three bipolar dorsal kineties; freshwater habitat.

**FIGURE 3:**
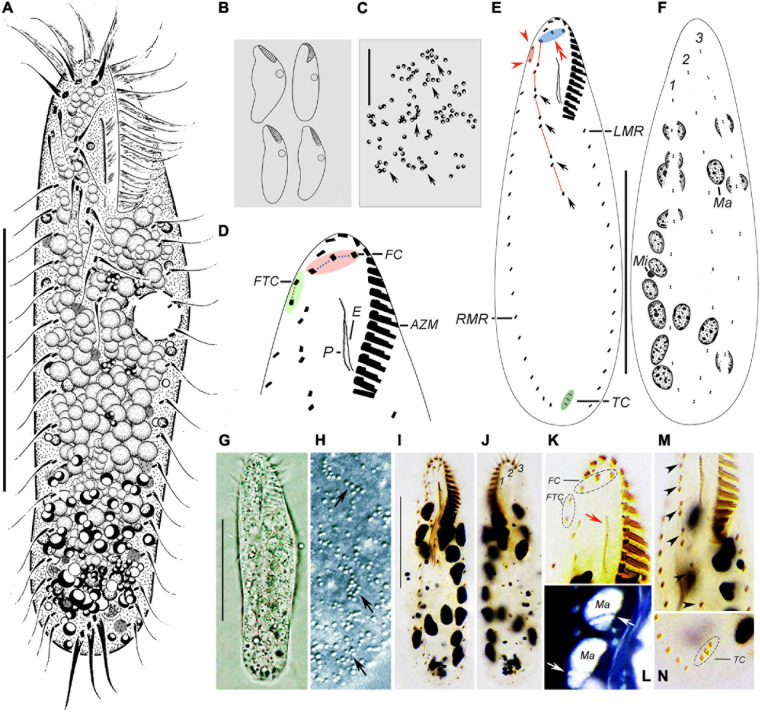
Morphology of *Wilbertophrya sinica* n. sp. from life **(A–C,G,H)** and after staining with protargol **(D–F,I–N)**. **(A)** Ventral view of a representative individual. **(B)** Ventral views to show different body shapes. **(C)** Arrangement of cortical granules on dorsal side (arrows). **(D)** Ventral view to demonstrate ciliature in frontoventral area. **(E,F)** Ventral **(E)** and dorsal **(F)** views showing the infraciliature. Arrows mark midventral pairs, arrowheads point to frontoterminal cirri, and double arrowheads indicate frontal cirri. **(G)** Ventral view. **(H)** Arrangement of cortical granules (arrows) on dorsal side. **(I)** Ventral view of the holotype specimen to show ventral ciliature. **(J)** Dorsal view to show dorsal kineties and macronuclear nodules. **(K)** Ventral view of anterior portion to show the frontal and frontoterminal cirri, endoral, and paroral (arrow). **(L)** Dorsal view to show macronuclear nodules and replication bands (arrows) (the color of image was inverted using Photoshop). **(M)** Ventral view to show the midventral pairs (arrowheads). **(N)** Ventral view of posterior end showing transverse cirri. AZM, adoral zone of membranelles; CV, contractile vacuoles; E, endoral; FC, frontal cirri; FTC, frontoterminal cirri; LMR, left marginal row; Ma, macronucleus nodules; Mi, micronuclei; P, paroral; RMR, right marginal row; TC, transverse cirri; 1–3, dorsal kineties 1–3. Scale bars: **(A,E,F,G,I)** = 40 μm, **(C)** = 15 μm.

*Type material:* One protargol-stained slide (No. MJY2017042201-3) with the holotype specimen (Figure 3I) circled in ink and two paratype slides (No. MJY2017042201-1, 2) were deposited in the Laboratory of Protozoological Biodiversity and Evolution in Wetland, Shaanxi Normal University, China.

*Type locality:* Puddle in a forest in Bomi County, Linzhi (29°39′N; 94°21′E), Tibet, China.

*Etymology:* The species-group name *sinica* recalls that this species was first discovered in China.

*Description* (Figures 3A–N and Table 1).

**TABLE 1:** Morphometric data of *Wilbertophrya sinica* n. g., n. sp. (*Ws*, upper line) and *Bakuella xianensis* n. sp. (*Bx*, lower line).

**Characters**	**Species**	**HT**	**Min**	**Max**	**Mean**	**Med**	**SD**	**CV**	***n***
Body, length in μm	*Ws*	118	69	118	91.1	90	13.9	15.3	20
	*Bx*	103	84	129	109.6	110	13.8	12.6	18
Body, width in μm	*Ws*	38	14	38	22.1	20	6.7	30.1	20
	*Bx*	50	29	58	47.1	48	8.5	18.0	18
AZM, length in μm	*Ws*	31	16	31	25.1	26	4.8	19.1	20
	*Bx*	42	31	50	40.7	41	4.8	11.7	18
Adoral membranelles, number	*Ws*	17	15	18	16.7	17	0.9	5.6	20
	*Bx*	25	23	33	28.4	29	2.8	9.9	18
Frontal cirri, number	*Ws*	3	3	3	3.0	3	0	0	20
	*Bx*	3	3	3	3.0	3	0	0	18
Buccal cirri, number	*Bx*	1	1	1	1.0	1	0	0	18
Frontoterminal cirri, number	*Ws*	2	2	2	2.0	2	0	0	20
	*Bx*	5	4	6	4.8	5	0.8	16.8	17
Number of cirral pairs in midventral complex	*Ws*	4	4	5	4.4	4	0.5	11.5	15
	*Bx*	9	8	12	10.9	11	1.3	12.1	17
Cirri in ventral row 1, number	*Bx*	3	2	3	2.1	2	0.3	16.1	16
Cirri in ventral row 2, number	*Bx*	4	3	5	3.7	4	0.6	16.3	16
Cirri in ventral row 3, number	*Bx*	–	4	5	4.8	5	0.5	10.5	4
Transverse cirri, number	*Ws*	3	2	4	3.0	3	0.5	15.3	20
	*Bx*	4	3	5	4.3	4	0.6	13.7	17
Pretransverse cirri, number	*Bx*	1	1	1	1.0	1	0	0	12
Left marginal cirri, number	*Ws*	15	13	21	16.0	15	2.1	13.3	20
	*Bx*	23	22	33	26.9	27	3.6	13.3	17
Right marginal cirri, number	*Ws*	17	14	25	18.1	18	2.4	13.3	20
	*Bx*	28	24	38	31.8	32	3.6	11.2	17
Dorsal kineties, number	*Ws*	3	3	3	3.0	3	0	0	20
	*Bx*	3	3	3	3.0	3	0	0	18
Macronuclear nodules, number	*Ws*	16	13	19	15.2	15	1.3	8.6	20
	*Bx*	66	50	106	69.6	65	16.7	24.0	17

*All data are based on protargol-stained specimens.*

*AZM, adoral zone of membranelles; CV, coefficient of variation in %; DK, dorsal kineties; HT, holotype specimen; Max, maximum; Mean, arithmetic mean; Med, median; Min, minimum; N, number of cells measured; SD, standard deviation; –, data unavailable.*

Body size 50–115 μm × 15–35 μm *in vivo*. Cell outline variable, generally elliptical to elongate-elliptical, right cell margin slightly concave, left margin slightly convex; usually widest in front of mid-body; dorsoventrally flattened; slightly flexible but non-contractile (Figures 3A, B, G). Nuclear apparatus composed of about 15 ellipsoidal nodules and one to four, on average two, micronuclei up to 2 μm in diameter (Figures 3F, L). Contractile vacuole about 7 μm in diameter, located slightly ahead of mid-body near left body margin, contracting at intervals of about 7 s (Figures 3A, B). Cortical granules colorless, globular, about 1 μm across, irregularly distributed (Figures 3C, H, arrows). Cytoplasm colorless or grayish, often containing numerous densely packed lipid droplets (Figures 3A, G). Locomotion by moderately fast crawling on the bottom of Petri dish, occasionally jerking back and forth; when suspended in water, cells often swim continuously in circles.

Infraciliature as shown in Figures 3D–F,I–K,M,N. The adoral zone is composed of 15–18 membranelles, occupying about 1/4–1/3 of body length (Table 1). In living cells, cilia of distal membranelles about 12 μm in length, and those of proximal membranelles are about 6 μm long. The distal end of the adoral zone extends only slightly onto right body margin forming a question mark shape as in other urostylids. Paroral longer than endoral, both generally straight and optically intersect at their upper region (Figures 3D, E). Three slightly enlarged frontal cirri about 8 μm in length, rightmost one located behind the distal end of the adoral zone (Figure 3D, pink area, Figure 3E, double arrowheads). Two frontoterminal cirri behind the distal end of the adoral zone (Figure 3E, arrowheads); buccal cirrus lacking (Figures 3D, E); two to four slightly enlarged transverse cirri, up to 12 μm long *in vivo*. Pretransverse cirri absent. Midventral complex composed of four or five pairs of cirri, terminating ahead of mid-body (Figure 3E, arrows). Left and right marginal rows composed of 13–21 and 14–25 cirri, respectively, with the anterior end of the left marginal rows not curved rightward (Figure 3E).

Three dorsal kineties arranged in typical *Gonostomum* pattern, with bristles about 3 μm in length (Figures 3F,J).

### Description of *Bakuella xianensis* n. sp.

ZooBank registration.

*Bakuella xianensis* n. sp.: urn:lsid:zoobank.org:act:7C3FA85 3-C973-402B-824D-21F5337CB63E

*Diagnosis:* Body 115–150 μm × 40–65 μm *in vivo*, elliptical in outline; about 70 macronuclear nodules; contractile vacuole located ahead of mid-body; dark-brownish cortical granules arranged in groups, about 0.7 μm across; about 23–33 adoral membranelles; midventral complex composed of 8–12 cirral pairs, terminating at about mid-body; two or three short ventral rows continuous with midventral pairs; three frontal, four to six frontoterminal, and three to five fine transverse cirri; one right and one left marginal rows with 24–38 and 22–33 cirri, respectively; three bipolar dorsal kineties; freshwater habitat.

**FIGURE 4:**
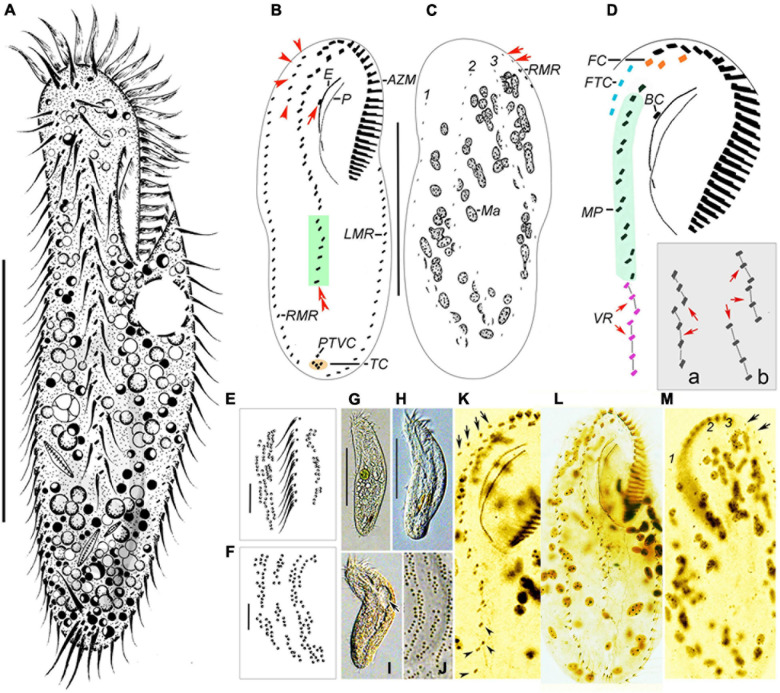
Morphology of *Bakuella xianensis* n. sp. from life **(A, E–J)** and after protargol staining **(B–D,K–M)**. **(A)** Ventral view of a representative specimen. **(B,C)** Ventral **(B)** and dorsal **(C)** views of the holotype specimen to show the infraciliature and nuclear apparatus. In **(B)**, arrow marks the buccal cirrus, arrowheads show the frontoterminal cirri, and double arrowheads mark a short ventral row (in green box). In **(C)**, arrows indicate the basal body pairs ahead of the right marginal row. **(D)** Ventral view to show details of buccal field and midventral complex; inset **(a,b)** shows two different arrangements of ventral rows (VR, arrows). **(E,F,J)** Detail of distribution of cortical granules in irregular longitudinal rows on ventral **(E)** and dorsal **(F,J)** sides. **(G–I)** Ventral views to show different body shapes due to flexibility of cells. Arrow indicates the contractile vacuole. **(K)** Ventral view to show details of buccal field and midventral complex, arrows show the frontoterminal cirri, and arrowheads indicate the midventral rows. **(L,M)** Ventral **(L)** and dorsal **(M)** views of the same specimen showing infraciliature and nuclear apparatus. Arrows indicate the basal body pairs ahead of right marginal row. AZM, adoral zone of membranelles; BC, buccal cirrus; CV, contractile vacuoles; E, endoral; FC, frontal cirri; FTC, frontoterminal cirri; LMR, left marginal row; Ma, macronuclear nodules; MVP, midventral pairs; P, paroral; PTVC, pretransverse ventral cirri; RMR, right marginal row; TC, transverse cirri; VR, ventral row; 1–3, dorsal kineties. Scale bars: **(A–C,G,H)** = 60 μm, **(E,F)** = 7 μm.

*Type material:* One protargol-stained slide (No. MJY2019052501-1) with the holotype specimen (Figure 4B) circled in ink and two paratype slides (No. MJY2019052501-2, 3, 4) were deposited in the Laboratory of Protozoological Biodiversity and Evolution in Wetland, Shaanxi Normal University, China.

*Type locality:* A freshwater pond in Chanba National Wetland Park, Xi’an (34°31′N; 109°01′E), China.

*Etymology:* The species-group name *xianensis* recalls that this species was first discovered in Xi’an, China.

*Description* (Figures 4A–M and Table 1).

Body about 115–150 μm × 40–65 μm *in vivo*, outline elongate-elliptical with both ends slightly narrow, length-to-width ratio approximately 3:1, flexible and dorsoventrally flattened (Figures 4A,G–I and Table 1). One contractile vacuole, about 15 μm across, positioned slightly in front of mid-body near left margin (Figures 4A,I, arrow). Pellicle thin and soft, with globular, dark-brownish cortical granules (about 0.7 μm across) in densely arranged groups on both ventral and dorsal sides, rendering cells slightly brownish at lower magnifications (Figures 4E, F, J). Cytoplasm colorless to grayish, usually packed with numerous small lipid droplets (about 3 μm across) and several large food vacuoles (about 8 μm across) containing ingested diatoms, bacteria, and/or ciliates. About 70 ellipsoidal macronuclear nodules scattered in cytoplasm (Figures 4C,M and Table 1). Locomotion by continuous, slow, to moderately fast, crawling on substrate.

Infraciliature as shown in Figures 4B–D,K–M. The adoral zone is about 40 μm long, occupying about 1/3 of body length, and composed of about 28 membranelles on average (Table 1). Endoral and paroral prominent *in vivo*, that is, long and curved, optically intersecting at the paroral’s mid-region. Most somatic cirri are relatively fine with cilia about 10–15 μm long. Constantly three relatively stout frontal cirri (FC, Figure 4D), apically positioned. Invariably one buccal cirrus, about level of endoral’s mid-region (Figure 4B, arrow). Four to six fine frontoterminal cirri (Figure 4B, arrowheads). Three to five transverse cirri form roughly V-shaped (Figures 4B,L), protruding beyond rear end of body. Cilia of which is 13–15 μm long. Of the 18 examined specimens, 12 had one pretransverse cirrus, while the remainder lacked the cirrus. Midventral complex terminates at about posterior 1/4 of body length, composed of about 8–12 cirral pairs forming a zigzag row that extends to about mid-region of the cell, and two, sometimes three, short ventral rows that are continuous with a row of midventral pairs (Figures 4B, D, VR and arrows, inset). One right and one left marginal rows composed of 24–38 and 22–33 cirri, respectively (Table 1); the right marginal row commences slightly ahead of level of buccal cirrus, always with two pairs of basal bodies ahead of its anterior end (Figures 4C,M, arrows). Three bipolar dorsal kineties arranged in typical *Gonostomum* pattern (Figures 4C,M).

### Phylogeny Based on Small Subunit Ribosomal DNA Sequence Data

**FIGURE 5:**
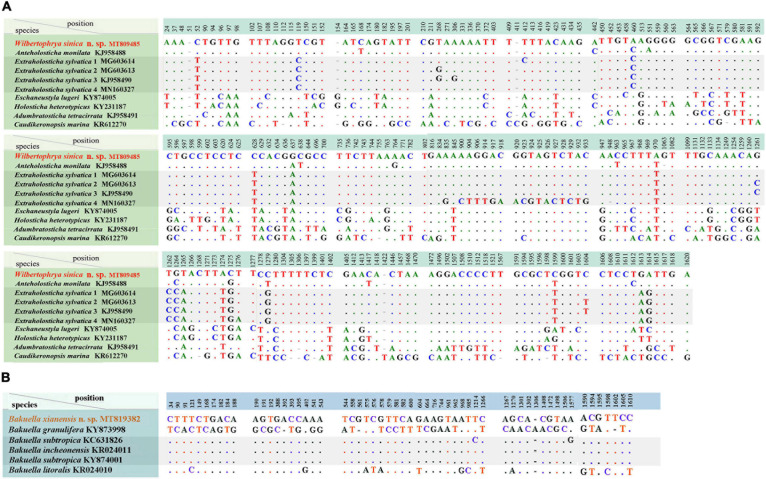
**(A)** Comparison of small subunit ribosomal DNA (SSU rDNA) sequences showing the unmatched nucleotides between *Wilbertophrya sinica* n. sp. and related species (for GenBank accession numbers, see Figure 2). **(B)** Comparison of SSU rDNA sequences showing the unmatched nucleotides between *Bakuella xianensis* n. sp. and related species (for GenBank accession numbers, see Figure 2). Nucleotide positions are given at the top of each column. Insertions and deletions are compensated by introducing alignment gaps (-). Matched sites are represented by dots (.).

The SSU rDNA sequences of *Wilbertophrya sinica* n. sp. and *Bakuella xianensis* n. sp. were deposited to GenBank with accession numbers, length, and guanine–cytosine (GC) content as follows: MT809485, 1,762 bp, 45.40% and MT819382, 1,770 bp, 44.18%, respectively. *W. sinica* and nine morphologically related isolates generated a nucleotide matrix with 204 unmatched sites (Figure 5A), while *B. xianensis* and five related taxa formed the table with 57 unmatched sites (Figure 5B).

Phylogenetic trees inferred from the SSU rDNA sequences using ML and BI had similar topologies; therefore, only the ML tree is shown with nodal support from both methods (Figure 2). *W. sinica* n. sp. clustered with *Anteholosticha monilata* (KJ958488) with high support (87% ML, 1.00 BI), which together grouped with four sequences of *Extraholosticha sylvatica* (MG603613, MG603614, KJ958490, and MN160327) with full support (Figure 2). This group was sister to the clade composed of *Eschaneustyla lugeri* (KY874005) and *Holostichides heterotypicus* (KY231187). Consequently, a comparison was made among these seven and two other morphologically similar species of *W. sinica* n. sp., namely, *Adumbratosticha tetracirrata* (KJ958491) and *Caudikeronopsis marina* (KR612270) (Figure 5A). The sequence differences between *W. sinica* n. sp. and these nine “related” taxa are as follows: 15 bp for *A. monilata*, 16–33 bp for the four sequences of *E. sylvatica*, 63 bp for *E. lugeri*, 67 bp for *H. heterotypicus*, 84 bp for *A. tetracirrata*, and 126 bp for *C. marina*.

*Bakuella xianensis* n. sp. clustered with *Bakuella incheonensis* (KR024011), and two sequences of *Bakuella subtropica* (KC631826 and KY874001) and *Anteholosticha paramanca* (KF806443) with moderate-to-strong support (90% ML, 1.00 BI), which together formed a clade with two species of *Anteholosticha*. Two other *Bakuella* species (*Bakuella litoralis* and *Bakuella granulifera*) belong to other groups, i.e., *B. litoralis* KR024010 + the *Neobakuella–Apobakuella–Diaxonella* complex and *B. granulifera* + *Urostyla* + *Neobakuella*. Hence, all known congeners in *Bakuella* belong to three separate (but neighboring) branches within the core-urostylid lineage (Figure 2).

## Discussion

### Systematic Position of *Wilbertophrya* n. g. and Comparison With Related Taxa

**FIGURE 6:**
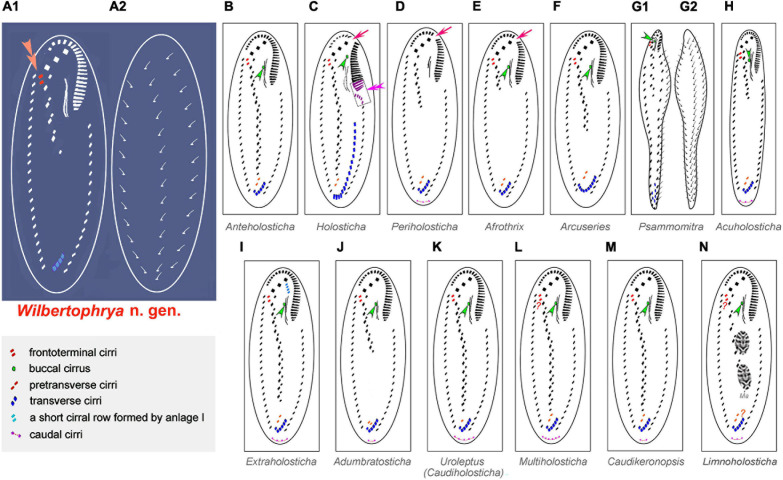
Comparison of infraciliature patterns of genera related to *Wilbertophrya* n. g. Arrows indicate the gap in the adoral zone of membranelles; arrowheads show the presence of a buccal cirrus. **(A1,A2)** Ventral **(A1)** and dorsal **(A2)** views of *Wilbertophrya* n. g.; double arrowheads in **(A)** indicate the frontoterminal cirri, while in **(C)**, they mark the curved anterior end of left marginal row. **(B)** *Anteholosticha*. **(C)** *Holosticha*. **(D)** *Periholosticha*. **(E)** *Afrothrix*. **(F)** *Arcuseries*. **(G1,G2)** ventral **(G1)** and dorsal **(G2)** views of *Psammomitra*. **(H)** *Acuholosticha*. **(I)** *Extraholosticha*. **(J)** *Adumbratosticha*. **(K)** *Caudiholosticha*. **(L)** *Multiholosticha*. **(M)** *Caudikeronopsis*. **(N)** *Limnoholosticha*. Ma, macronuclear nodules. Question mark in **(L)** indicates presence/absence of frontoterminal questionable. Question mark in **(N)** indicates presence/absence of frontoterminal and pretransverse ventral cirri not known.

The possession of three clearly differentiated frontal cirri and a midventral complex composed of midventral pairs only places *Wilbertophrya* n. g. unequivocally in the family Holostichidae (*sensu* Berger, 2006). Based on the following combination of features, that is, single left and right marginal rows, a continuous adoral zone, presence of frontoterminal cirri, clearly differentiated frontal cirri, and midventral cirral pairs arranged in a zigzag pattern, five genera in the family Holostichidae should be compared with *Wilbertophrya* n. g., namely, *Anteholosticha* Berger, 2003; *Arcuseries* Huang et al., 2014; *Afrothrix* Foissner, 1999; *Acuholosticha* Li et al., 2017; and *Holosticha* Wrzesniowski, 1877 (Berger, 2003; Huang et al., 2014; Li et al., 2017). All five of these genera differ from *Wilbertophrya* n. g. in having a buccal cirrus and pretransverse cirri, whereas both these structures are lacking in the new genus (Berger, 2006; Huang et al., 2014; Li et al., 2017). Furthermore, *Acuholosticha* possesses caudal cirri, whereas these are absent in *Wilbertophrya* n. g. (Berger, 2006; Figure 6; Table 2). The validity of the new genus is also supported by the molecular data (Figure 5A).

**TABLE 2:** Morphological comparison of *Wilbertophrya* n. g. with similar genera.

**Character Genera**	**Buccal cirrus**	**Pretransverse ventral cirri**	**Caudal cirri**	**Adoral zone of membranelles**	**Number of dorsal kineties**	**Frontoterminal cirri**	**Other structures**	**Data source**
*Acuholosticha*	Present	Present or absent	Present	Continuous	2–4	Present		Li et al., 2017
*Adumbratosticha*	Present	Present or absent	Present	Continuous	More than 3	Present		Li et al., 2017
*Afrothrix*	Present	Present	Absent	Bipartite	2–3	Present		Berger, 2006
*Anteholosticha*	Present	Present or absent	Absent	Continuous	3–6	Present		Berger, 2006
*Arcuseries*	Present	Present	Absent	Continuous	3	Present		Huang et al., 2014
*Caudikeronopsis*	Present	Present	Present	Continuous	More than 3	Present		Li et al., 2017
*Extraholosticha*	Present	Present	Present	Continuous	More than 3	Present	A short cirral row formed by anlage I	Li et al., 2017
*Holosticha*	Present	Present	Absent	Bipartite	More than 3	Present	Rear membranelles of proximal portion slightly to distinctly wider than remaining; anterior end of left marginal row distinctly curved rightward; number of transverse cirri equal to or only slightly lower than number of midventral pairs	Berger, 2006
*Limnoholosticha*	Present	NA	Present	Continuous	NA	NA		Li et al., 2017
*Multiholosticha*	Present	Present	Present	Continuous	6	NA		Li et al., 2017
*Periholosticha*	Absent	NA	Present	Bipartite	3 or 2	Present or absent		Berger, 2006
*Psammomitra*	Present	NA	Absent	Continuous	4	Present	Body tripartite in head, trunk, and tail	Berger, 2006
*Uroleptus*	Present	Absent	Present	Continuous	5	Present		Li et al., 2017
*Wilbertophrya* n. g.	Absent	Absent	Absent	Continuous	3	Present		Present work

*NA, not available.*

Eight other morphologically similar genera are compared with *Wilbertophrya* n. g., namely, *Adumbratosticha* Li et al., 2017; *Caudikeronopsis* Li et al., 2017; *Extraholosticha* Li et al., 2017; *Limnoholosticha* Li et al., 2017; *Multiholosticha* Li et al., 2017; *Periholosticha* Hemberger, 1985; *Psammomitra* Borror, 1972; and *Uroleptus* Ehrenberg, 1831 (Figure 6; Table 2). *Wilbertophrya* n. g. can be separated from each of these by the following combination of characters: (1) presence of frontoterminal cirri; (2) absence of pretransverse, caudal, and buccal cirri; (3) adoral zone of membranelles continuous; and (4) three complete dorsal kineties (Figure 6; Table 2).

The systematic position of *Wilbertophrya* n. g. remains unclear considering that the family assignment though morphological information supports its possible assignment to the family Holostichidae. The SSU rDNA tree reveals that *Wilbertophrya* groups with seven representatives of four genera: *Anteholosticha*, *Extraholosticha*, *Eschaneustyla*, and *Holostichides* (Figure 2). It is most closely related to *A. monilata* (KJ958488), the identification of which needs to be verified since there is another sequence with the same name but which occupies a different position in the tree (Figure 2, double arrowheads). Previous molecular phylogenetic analyses suggest that the genus *Anteholosticha* is not monophyletic and that most of its nominal species belong to the Urostylidae + Pseudokeronopsidae + Pseudourostylidae complex, which is the sister group of the clade that includes *Wilbertophrya* (Figure 2; Shao et al., 2011; Li et al., 2017; Luo et al., 2018; Xu et al., 2020).

In the SSU rDNA tree (Figure 2), the new genus clusters with several representatives of the genus *Extraholosticha* followed by *E. lugeri* + *H. heterotypicus*. This large clade is separated from other sequences at a deep level, suggesting that it might represent an undefined group at about family level. In terms of its morphology, however, the genus *Wilbertophrya* should be assigned to the family Holostichidae (see above). But as revealed in previous molecular studies, the Holostichidae complex (e.g., Holostichidae + Urostylidae + Pseudokeronopsidae) is a so-called melting pot of taxa, the evolutionary relationships of which cannot be resolved using phylogenetic analyses of single-gene sequence data (Bernhard et al., 2001; Yi and Song, 2011; Zhao et al., 2015; Luo et al., 2018; Jung and Berger, 2019; Paiva, 2020; Wang et al., 2021b; Xu et al., 2020). Similarly, it is difficult to place *Wilbertophrya* n. g. into any known family based on SSU rDNA sequence data. Since ontogenetic information is unavailable for the new genus, we suggest that *Wilbertophrya* n. g. should be regarded as *incertae sedis* within the order Urostylida pending the availability of further data.

*Wilbertophrya* n. g. is a monotypic genus, and thus, the type species, *W. sinica* n. sp., can be separated from its most “similar” morphospecies, that is, members of the genera *Adumbratosticha*, *Periholosticha*, *Afrothrix*, and *Acuholosticha* (see Figure 6) by the same combination of features that define the genus, i.e., the absence of the buccal cirrus and pretransverse cirri, and the presence of sparsely distributed cortical granules and the conspicuously short midventral rows (Berger, 2003; Li et al., 2017; Shao et al., 2020). The validity of *W. sinica* n. sp. as a distinct species is also firmly supported by the molecular data, which demonstrates its considerable difference from other taxa (Figure 5A).

### Systematic Position of *Bakuella xianensis* n. sp. and Comparison With Congeners

The well-defined genus *Bakuella* can be recognized by its midventral complex comprising pairs of cirri arranged in a zigzag pattern and several obliquely oriented fragment-like ventral rows (Borror and Wicklow, 1983; Berger, 2006; Song and Shao, 2017; Shao et al., 2020). To date, 12 nominal species have been reported (Table 3), three of which have a single buccal cirrus and more than two frontoterminal cirri, and so should be compared with *B. xianensis* n. sp., namely, *Bakuella agamalievi* Borror and Wicklow, 1983; *B. subtropica* Chen et al., 2013; and *B. incheonensis* Jo et al., 2015.

**TABLE 3:** Morphological comparison of *Bakuella xianensis* n. sp. with closely related species.

**Character Species**	**Body length *in vivo* (μm)**	**AZM, no.**	**Buccal cirri, no.**	**Frontoterminal cirri, no.**	**Midventral pairs, no.**	**Midventral rows, no.**	**Midventral complex, length**	**Transverse cirri, no.**	**Cirri in LMR, no.**	**Cirri in RMR, no.**	**Cortical granules**	**Data source**
*Bakuella agamalievi*	100–150	26–37	1	4–7	9–18	3–6	Terminates bout 67% down length of body	4–7	30–40	34–47	0.8 μm across; colorless or slightly greenish	Berger, 2006
*Bakuella crenata*	About 210	28–30	3 or 4	7–9 (data from illustration)	About 5	10–12	Terminates slightly ahead of transverse cirri	7–9	36–40	40–50	NA	Berger, 2006
*Bakuella edaphoni*	190–300	34–45	5–9	2–5	5–14	5–10 with more than 3 cirri and 1–6 with 3 cirri	Terminating close to transverse cirrus	6–11	44–56	43–55	Absent	Berger, 2006
*Bakuella granulifera*	270–400	44–62	7–10	3–5	12–23	3–5	Terminates at right transverse cirrus	9–15	54–77	54–76	1.3–1.5 μm × 0.8–1.0 μm; brilliant citrine	Berger, 2006
*Bakuella incheonensis*	70–105	21–25	1	3 or 4	7–10	1 or 2	Terminates bout 62% down length of body	4 or 5	20–28	25–32	0.7 μm across; yellowish	Jo et al., 2015
*Bakuella marina*	About 200	28–51	2–5	5–11	4–12	4–8	Extends almost to transverse cirral row	5–11	23–56	34–63	NA	Berger, 2006
*Bakuella nilgiri*	About 158	42–54	4–8	2–4	18–23	2–10	Extends to near transverse cirri	6–11	38–56	49–65	About 0.7 μm across; colorless	Kumar et al., 2010
*Bakuella pampinaria oligocirrata*	80–150	22–34	2–5	1–7	3–7	3–5	Terminates very close to right transverse cirrus	3–7	24–42	25–42	About 1.0 μm × 0.7 μm; citrine to yellowish	Berger, 2006
*Bakuella pampinaria pampinaria*	90–180	22–39	3–6	5–8	6–13	2–6	Terminates very close to right transverse cirrus	2–5	24–47	23–51	1.5–2.0 μm × 1.0–1.5 μm; yellowish	Berger, 2006
*Bakuella subtropica*	100–150	25–44	1	4–12	9–23	1 or 2	Terminates bout 80% down length of body	3–6	30–54	28–64	1–2 μm across; yellow-brownish to yellow-greenish	Chen et al., 2013
*Bakuella xianensis* n. sp.	115–150	23–33	1	4–6	8–12	2 or 3	Terminates bout 75% down length of body	3–5	22–33	24–38	about 0.7 μm cross; dark-brownish	Present work

*AZM, adoral zone of membranelles; LMR, left marginal row; NA, not available; RMR, right marginal rows.*

**FIGURE 7:**
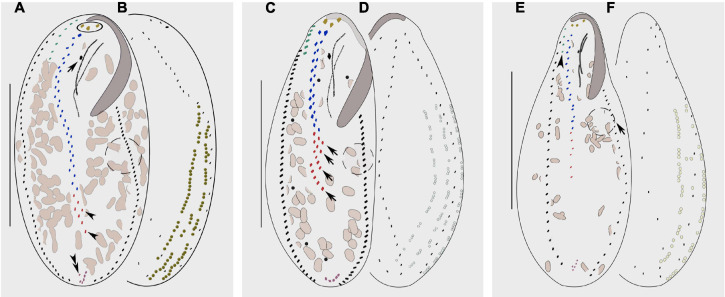
Morphology of *Bakuella subtropica* **(A,B)**, *Bakuella agamalievi* **(C,D)**, and *Bakuella incheonensis* **(E,F)**. **(A,C,E)** Ventral view of three individuals. **(B,D,F)** Dorsal views of three individuals and cortical granules on dorsal side. **(A)** Ventral view to show the infraciliature; circle marks the frontal cirri, arrow shows the buccal cirrus, arrowheads indicate the ventral rows, and double arrowheads show the pretransverse cirrus. **(B)** Distribution of cortical granules, which form longitudinal rows. **(C)** Ventral view of infraciliature; arrows mark the short ventral rows. **(D)** Distribution of cortical granules, typically grouped together and sparsely arranged in short longitudinal rows. **(E)** Ventral view of infraciliature, arrow marks the contractile vacuole, and arrowhead indicates the frontoterminal cirri. **(F)** Distribution of cortical granules, mainly distributed along cirral rows and dorsal kineties. Scale bars: **(A–D)** = 60 μm, **(E,F)** = 40 μm.

*Bakuella xianensis* n. sp. can be distinguished from *B. subtropica*, a brackish water form originally found in a subtropical area in China (estuary of the Pearl River, Chen et al., 2013; Figures 7A, B) by (1) the size of the cortical granules (about 0.7 μm in diameter vs. 1–2 μm in diameter); (2) its habitat (freshwater vs. brackish water in mangrove wetlands); (3) having fewer cirral pairs in the midventral complex (8–12 vs. 9–23); and (4) having fewer left (22–33 vs. 30–54) and right (24–38 vs. 28–64) marginal cirri (Chen et al., 2013).

*Bakuella xianensis* n. sp. differs from *B. agamalievi*, a marine form originally found in the Caspian Sea (Agamaliev, 1972; Figures 7C, D) and redescribed by Song et al. (2002) as having dark-brownish (vs. colorless or slightly greenish) cortical granules, fewer ventral rows in the midventral complex (two or three vs. three to six), fewer cirri in both right (24–38 vs. 40–47) marginal rows, and in its freshwater (vs. marine) habitat (Agamaliev, 1972; Song et al., 2002; Berger, 2006).

*Bakuella xianensis* n. sp. differs from *B. incheonensis* (Figures 7E, F) by its larger body size (115–150 μm × 40–65 μm vs. 70–105 μm × 20–40 μm *in vivo*), the color and arrangement of cortical granules (dark-brownish vs. yellowish), and its freshwater (vs. marine) habitat (Figures 4E, F, J, 7E, F). In addition, the cortical granules in *B. incheonensis* appear to be large, conspicuous, and possibly ellipsoid in shape (Figure 7F, arrows, not mentioned in original report), whereas those in *B. xianensis* n. sp. are small, globular, and inconspicuous (Figures 4E, F, J; Jo et al., 2015).

Including *B. xianensis* n. sp., SSU rDNA sequence data are available for only six species of *Bakuella*. These are grouped into three neighboring clades in the SSU rDNA tree (Figure 2). *B. xianensis* n. sp. is placed within the core clade of the genus. It is noteworthy, however, that some *Bakuella* sequences group with non-bakuellid genera, e.g., nominal isolates of genera such as *Urostyla*, *Diaxonella*, and *Anteholosticha*, with high support. This is consistent with previous reports (Chen et al., 2010, 2013) and indicates that none of the families Bakuellidae, Urostylidae, and Holostichidae is monophyletic; and the systematics of each requires further investigation.

## Data Availability Statement

The datasets presented in this study can be found in online repositories. The names of the repository/repositories and accession number(s) can be found in the article/supplementary material.

## Author Contributions

JM carried out the live observation and protargol impregnation. TZ was responsible for DNA amplification and sequencing, and the molecular phylogenetic analyses. JM, TZ, and CS performed the manuscript draft. CS and WS performed the manuscript review and editing. All authors helped to revise the manuscript, and read and approved the final manuscript.

## Conflict of Interest

The authors declare that the research was conducted in the absence of any commercial or financial relationships that could be construed as a potential conflict of interest.

## Publisher’s Note

All claims expressed in this article are solely those of the authors and do not necessarily represent those of their affiliated organizations, or those of the publisher, the editors and the reviewers. Any product that may be evaluated in this article, or claim that may be made by its manufacturer, is not guaranteed or endorsed by the publisher.

## Footnotes

1
http://guidance.tau.ac.il/
